# Spatio-Temporal Graph Neural Networks for Anomaly Detection in Complex Industrial Processes

**DOI:** 10.3390/s26051597

**Published:** 2026-03-04

**Authors:** Shutian Zhao, Hang Zhang, Bei Sun, Yijun Wang

**Affiliations:** School of Automation, Central South University, Changsha 410083, China; 18209581688@163.com (S.Z.); zhang22@csu.edu.cn (H.Z.); sunbei@csu.edu.cn (B.S.)

**Keywords:** anomaly detection, spatio-temporal modeling, linear attention, industrial processes, variational autoencoder

## Abstract

With the advancement of intelligent manufacturing strategies, Cyber–Physical Production Systems (CPPSs) generate massive amounts of multidimensional, dynamic, and non-stationary data, posing significant challenges to real-time Process Monitoring. Existing anomaly detection methods often suffer from insufficient feature robustness when dealing with complex spatio-temporal dynamics, high computational complexity, and difficulties in effectively capturing incipient faults within deep topological structures. To address these issues, this paper proposes a **Spatio-Temporal Variational Graph Statistical Attention Autoencoder (ST-VGSAE)**. First, the framework performs end-to-end multi-scale temporal decomposition via an Adaptive Lifting Wavelet Module, which enhances feature robustness while effectively suppressing noise. Furthermore, a spatio-temporal Token statistical self-attention mechanism with linear complexity is incorporated. By modulating local features via global statistics, it significantly reduces computational costs while enhancing anomaly discriminability. Experiments on the Tennessee Eastman (TE) process dataset demonstrate that the proposed model significantly outperforms state-of-the-art methods in key metrics such as the Fault Detection Rate and the False Alarm Rate, exhibiting superior noise robustness and real-time performance.

## 1. Introduction

Under the deep advancement of intelligent manufacturing strategies, the modern manufacturing industry is undergoing a profound paradigm shift from automation to autonomy, giving birth to highly integrated Cyber–Physical Production Systems (CPPSs) [1,2]. These systems encompass numerous critical domains, characterized fundamentally by the deep integration of physical entities and digital spaces through densely distributed sensor networks [3]. The data streams generated by this convergence are characterized not only by high Volume and Velocity but also by unprecedented Variety, dynamic Veracity, and Variability [4,5]. In this macro context, real-time Process Monitoring and anomaly detection are no longer merely auxiliary operational tools; they have become absolute prerequisites for ensuring production safety, optimizing operational efficiency, guaranteeing product quality, and enabling Predictive Maintenance (PdM) [6,7]. Consequently, there is an urgent need to develop solutions capable of efficiently processing industrial data with complex spatio-temporal characteristics to cope with the increasingly rigorous operational environments and data challenges [8].

Data-driven process monitoring methods have witnessed substantial development over the past few decades, evolving into a landscape where Multivariate Statistical Process Monitoring (MSPM) and Deep Learning advance in parallel [9]. MSPM techniques, particularly Principal Component Analysis (PCA) and its variants, have played a foundational role in handling high-dimensional industrial data [10]. To address the non-stationarity prevalent in modern CPPS, Recursive PCA (RPCA) has seen significant improvements [11]; notably, Søndergaard et al. (2024) introduced Individual Contextual Forgetting Factors, enabling adaptive models to intelligently distinguish between transient disturbances and genuine operating condition shifts, thereby effectively resolving the stability–plasticity dilemma in adaptive algorithms [12]. Addressing serial correlations in dynamic processes, Dynamic PCA (DPCA) has evolved beyond mere feature extraction. Zheng et al. (2024) proposed Dynamic Control PCA (DCPCA), which further established an algebraic mapping between latent variables and control variables, realizing closed-loop control from fault detection to automatic recovery [13]. Similarly, to address the adaptability in dynamic environments, Liang et al. (2023) developed an enhanced PI controller based on adaptive iterative learning control, effectively optimizing the system’s robust performance under non-linear constraints [14]. Furthermore, targeting strong process nonlinearity, the Reduced Kernel PCA (RKPCA) proposed by Attouri et al. (2024) successfully overcame the computational bottlenecks of kernel methods on large-scale datasets via spectral clustering and random sampling strategies [15]. In terms of enhancing model robustness and interpretability, Zheng & Mak (2024) extended robust PCA to exponential family distributions to accommodate non-Gaussian data [16], while Zhang et al. (2025) improved the feature selection capability of sparse PCA using the Harris Hawks Optimization algorithm [17].

Concurrently, breakthroughs in Deep Learning technologies have provided powerful tools for modeling complex nonlinearities and spatio-temporal dependencies [18,19]. In temporal modeling, Lachekhab et al. (2024) demonstrated the advantages of a hybrid LSTM–Autoencoder architecture in capturing long-term dependencies in motor vibration signals, with a reconstruction-error-based detection mechanism significantly outperforming traditional statistical methods [20]. To overcome the serial computation limitations of RNNs and capture multi-scale features, Varalakshmi & Lingaraju (2024) successfully applied Temporal Convolutional Networks (TCNs) to motor and acoustic fault diagnosis, utilizing dilated causal convolutions to achieve efficient real-time inference [21]. In the field of generative models, Komorska & Puchalski (2024) constructed equipment health indices using the continuous latent space of Variational Autoencoders (VAEs), bridging the gap between discrete fault detection and continuous degradation monitoring [22]. Advancing this domain further, Lv et al. (2025) proposed an incremental variational graph attention Autoencoder that utilizes probabilistic inference to achieve adaptive and interpretable process monitoring [23]. In terms of multi-modal and visual inspection, recent work has also introduced a multi-expert diffusion model for surface defect detection in specialized equipment, broadening the application of generative AI in industrial scenarios [24]; meanwhile, the MFGAN framework proposed by Qu et al. (2024) achieved robust multi-modal fusion of visual and sensor data via attention mechanisms [25]. Finally, given the topological network characteristics of industrial systems, Graph Neural Networks (GNNs) have emerged as a current research hotspot. In this context, cross-disciplinary approaches, such as integrating transformer architectures to dynamically learn graph structures, have demonstrated immense potential in capturing complex variable dependencies for multivariate time-series anomaly detection [26]. The Multi-Scale Dynamic Graph Neural Network (MSDG) proposed by Zhao et al. (2024) dynamically constructs dependency graphs between variables via a sliding window mechanism, precisely capturing the evolution of spatio-temporal correlations [27]. Hou et al. (2024) utilized the variation in attention weights within Graph Attention Networks (GATs) to localize fault propagation paths, significantly enhancing the interpretability of root cause analysis [28]. Furthermore, Gao et al. (2025) proposed D-GATAD, which is capable of capturing global dependencies across the entire plant [29]. Recent breakthroughs also focus on complex topological dynamics: Lv et al. (2025) introduced a hierarchical stochastic network approach for diagnosing faults in complex processes [30]; moreover, cutting-edge research has explored self-perturbed graph dynamics for multivariate time-series anomaly detection (2025) [31], as well as a Mixture-of-Experts framework (MoEGAD) with pseudo-anomaly generation for graph-level detection (2026) [32], significantly enhancing feature robustness against irregularities.

Despite the significant progress achieved by the aforementioned methods, critical challenges remain when monitoring complex, especially large-scale, CPPS: **(1) Insufficient feature robustness under complex temporal dynamics:** Industrial process data often exhibit complex multi-scale temporal dynamics and transient changes. Existing methods, when extracting features from raw signals, often struggle to distinguish between normal dynamic evolution and genuine anomalous fluctuations [9,10]. **(2) Limited capability in capturing incipient fault features under complex topological structures:** Traditional graph aggregation methods primarily focus on local neighborhood structures, often lacking awareness of the global graph distribution [33]. This limitation restricts the network’s ability to identify nodes that are structurally normal but attributively anomalous, thereby constraining the model’s performance in detecting subtle, incipient faults. **(3) Dilemma between computational efficiency and representation optimization in attention mechanisms:** The computational complexity of attention mechanisms typically grows quadratically with the number of nodes [34]. Moreover, existing attention mechanisms lack explicit optimization objectives to guide the learning of efficient representations, making it difficult to achieve a theoretical balance between “inter-group separability” and “intra-group compactness” [35].

To address these challenges, this paper proposes a **Spatio-Temporal Variational Graph Statistical Attention Autoencoder (ST-VGSAE)**. This framework combines the advantages of multi-scale temporal feature extraction, variational inference, and linear-complexity graph attention operators. The main contributions of this paper are summarized as follows:

(1) An **adaptive multi-scale temporal feature extraction module** is introduced to realize end-to-end multi-scale temporal feature learning. This module adaptively decomposes and extracts dynamic features from industrial data, improving feature robustness under complex temporal dynamics. (2) A **graph attention operator with linear complexity** is designed based on a statistical self-attention mechanism. This not only resolves the computational bottleneck in large-scale system monitoring but also significantly strengthens model interpretability, effectively supporting fault localization and variable contribution analysis. (3) By seamlessly integrating the aforementioned temporal and spatial features, the **ST-VGSAE model** is proposed. Through the fusion of multi-scale temporal feature extraction, deep graph learning, and efficient attention mechanisms, it achieves precise modeling of the spatio-temporal dynamics of complex industrial processes.

## 2. Materials and Methods

**Figure 1:**
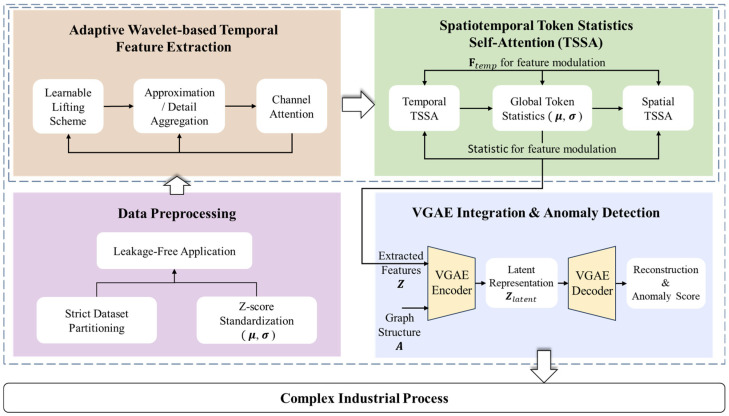
The architecture of the proposed model.

In this section, we delineate the proposed fault detection framework for industrial processes, predicated on **Adaptive Wavelets** and **Dynamic Graph Neural Networks (DGNNs)**. By integrating an Adaptive Lifting Wavelet scheme and a **Spatio-temporal Statistical Self-Attention** mechanism, the framework effectively addresses the challenges of non-stationarity, long-term dependencies, and complex spatial topologies inherent in industrial data. The implementation details and architectural innovations are summarized as follows (Figure 1):

(1) Data Preprocessing and Benchmark Construction

This study utilizes the TE benchmark dataset, which encompasses one normal operating condition and 28 distinct fault patterns. To eliminate dimensional disparities, the data are first standardized using the Z-score method. We maintain experimental rigor by strictly partitioning the data into a training set (for offline optimization), a validation set (for threshold determination), and a testing set (for online evaluation).

(2) Temporal Feature Extraction

To mitigate temporal noise, a learnable module based on **Adaptive Lifting Wavelets** is introduced to dynamically optimize prediction and update operators. The module decomposes signals into approximation and detail components. Long-term trends are preserved through final-moment aggregation of the approximation components, while high-frequency anomalous fluctuations are captured via energy pooling of the detail components. This module effectively transforms variable-length time series into compact, discriminative node features, serving as robust inputs for the VGAE.

(3) Joint Spatio-temporal Statistical Self-Attention Modeling

The framework employs a cascaded architecture with linear complexity. In the temporal dimension, a “denoise-then-focus” strategy is implemented, where TSSA captures trend anomalies following wavelet-based noise reduction. Spatially, a hybrid **GCN-TSSA** encoder is constructed to modulate local features using global graph statistics, significantly bolstering feature discriminability through global gradient coupling effects.

(4) Anomaly Detection and Evaluation

Online detection is performed by calculating anomaly scores based on **reconstruction errors** (integrating both feature and structural discrepancies). To address the limitations of static thresholds, an adaptive thresholding method based on **Gaussian Kernel Density Estimation (KDE)** is introduced. The model’s performance is comprehensively evaluated using a multi-dimensional metric suite, including the False Alarm Rate (FAR), the Fault Detection Rate (FDR), and Accuracy.

### 2.1. Data Preprocessing

To validate the effectiveness of the proposed methodology, fault detection experiments were conducted on the TE process benchmark. The TE dataset comprises 52 process variables across one normal state and 21 fault modes. Preprocessing involves two primary stages: data standardization and dataset partitioning.

#### 2.1.1. Data Standardization

Given the significant variations in scale and magnitude among process variables collected by different sensors, raw data may lead to training instability or slow convergence. Consequently, this study employs the Z-score method for standardization. Specifically, the mean μ and standard deviation σ for each feature variable are calculated using the training data under normal conditions. The standardization formula is defined as

(1) $$x_{scaled}=\frac{x-\mu }{\sigma },$$

where x represents the raw observation and $x_{scaled}$ denotes the standardized value. To strictly adhere to the causal constraints inherent in industrial scenarios and to prevent **data leakage**, the validation and testing sets are standardized using the statistical parameters (μ and σ) derived exclusively from the training set. Post-processing, each feature exhibits a mean of zero and one standard deviation, effectively eliminating dimensional bias and enhancing the convergence efficiency of the model training process.

#### 2.1.2. Dataset Partitioning

In this study, the TE dataset was partitioned into the training, validation, and test sets. The specific allocation strategies are detailed as follows:

**Training Set**: A total of 500 samples under normal operating conditions were selected for offline model training and parameter optimization. **Validation Set**: The remaining normal samples were utilized to determine the control limits or thresholds for fault detection. **Test Set**: This set was employed to evaluate the online monitoring performance of the proposed model, comprising both normal and faulty data. **Normal Test Set**: Consisting of 960 normal samples, this subset was used to verify the reconstruction error and the False Alarm Rate (FAR) in the absence of faults. **Faulty Test Set**: This subset encompasses 21 distinct fault modes (Fault 1 to Fault 21). Each fault mode contains 960 samples, where the first 160 samples represent normal operation, and the fault is introduced from the 161st sample onwards (resulting in 800 faulty samples per mode). This configuration was designed to assess the Fault Detection Rate (FDR) and detection latency of the model.

### 2.2. Adaptive Wavelet-Based Temporal Feature Extraction

**Figure 2:**
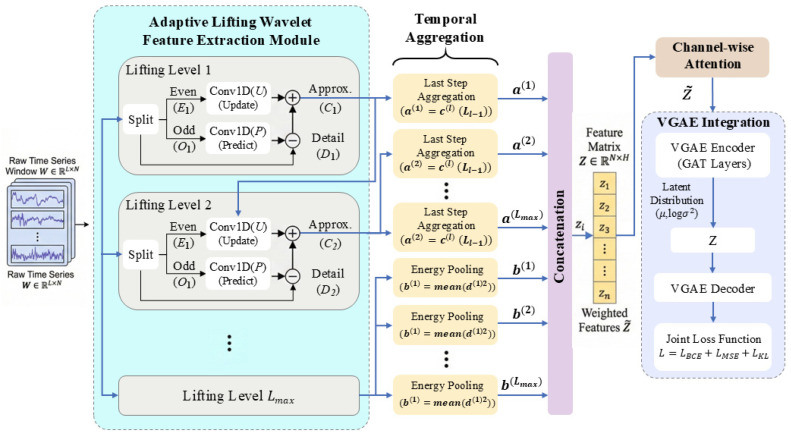
Adaptive wavelet transform [36].

To address the limitations of conventional Graph Neural Networks (GNNs) in terms of robustness against non-stationarity when processing raw time windows directly and to effectively capture multi-scale temporal dependencies, an input-level temporal feature extraction module based on the **Adaptive Lifting Wavelet** is integrated into the **VGAE** framework. This module is designed to map high-dimensional, noisy time-series windows $W\in R^{L\times N}$ into compact node feature matrices $Z\in R^{N\times H}$ that are rich in discriminative information. The detailed processing steps are as follows (Figure 2):

#### 2.2.1. Learnable Lifting Scheme Framework

In contrast to conventional wavelet transforms that employ fixed basis functions, this study adopts the **Lifting Scheme**—often referred to as the “second-generation wavelet.” This approach utilizes a data-driven mechanism to learn **prediction** and **update operators**, thereby adapting to specific temporal patterns. For the input sequence x of an arbitrary node i, a multi-level decomposition structure is implemented. At the l-th level of decomposition, the input signal $X^{\left(l-1\right)}$ is first partitioned (**Split**) into even and odd sequences, denoted as $E^{\left(l\right)}$ and $O^{\left(l\right)}$, respectively. Subsequently, a trainable 1D convolutional neural network is employed to implement the prediction operator $\Phi_{P}^{\left(l\right)}$ and the update operator $\Phi_{U}^{\left(l\right)}$. This iterative process generates the **detail coefficient** $D^{\left(l\right)}$ and the **approximation coefficient** $C^{\left(l\right)}$ as follows:

(2) $$C^{\left(l\right)}=E^{\left(l\right)}+\Phi_{U}^{\left(l\right)}\left(O^{\left(l\right)}\right)$$

(3) $$D^{\left(l\right)}=O^{\left(l\right)}-\Phi_{P}^{\left(l\right)}\left(C^{\left(l\right)}\right)$$

In this framework, $X^{\left(l\right)}=C^{\left(l\right)}$ serves as the input for the subsequent decomposition level. This process is executed iteratively until the predefined maximum decomposition level $L_{\max}$ is reached. Such a design enables the model to perform multi-scale decomposition directly in the time domain, thereby simultaneously capturing local transients and long-term trends of the signal. For a single node, the time series is represented as a discrete signal $x=\{x(t){\}}_{t=0}^{L-1}\in R^{L}$, the wavelet transform expands the signal into a family of scaled and translated versions of the mother wavelet $\psi_{j,k}$ across multiple scales j and shifts k:

(4) $$x(t)\approx \sum_{j\in J}\;\sum_{k\in K_{j}}\;c_{j,k}\psi_{j,k}(t),c_{j,k}=\langle x,\psi_{j,k}\rangle ,$$

where t denotes the time index; j represents the scale (where larger scales correspond to lower-frequency components); k is the time translation index at a given scale; $\psi_{j,k}(t)$ is the wavelet basis function at scale j and shift k; and $c_{j,k}$ are the corresponding wavelet coefficients, reflecting the signal energy at that specific scale and temporal location.

Unlike the Fourier transform, which expands signals solely in the frequency domain, the aforementioned formulation encodes both temporal and spectral information through localized wavelet basis functions. This makes the approach significantly more sensitive to local abrupt changes, trend variations, and periodic disturbances within non-stationary time series.

#### 2.2.2. Temporal Aggregation of Approximation and Detail Components

To transform variable-length temporal components into fixed-dimensional features suitable for graph-based models and to highlight key information essential for anomaly detection, we apply specific aggregation methods and pooling strategies to the approximation and detail sequences of node i at level l, denoted as $c_{i}^{\left(l\right)}(t)=C_{i,1,t}^{\left(l\right)}$ and $d_{i}^{\left(l\right)}(t)=D_{i,1,t}^{\left(l\right)}$ for $t=0,\ldots ,L_{l}-1$:

**Last-moment Aggregation for Approximation Components**: For the approximation component $c_{i}^{\left(l\right)}(t)$, which reflects long-term trends, we employ last-moment sampling

(5) $$a_{i}^{\left(l\right)}=c_{i}^{\left(l\right)}(L_{l}-1)$$

to preserve the low-frequency baseline information of the signal.

**Energy Pooling for Detail Components**: For the detail component $d_{i}^{\left(l\right)}(t)$, which characterizes high-frequency disturbances, this study proposes an **Energy Pooling** strategy. Given that anomalies often manifest as high-frequency fluctuations, Energy Pooling effectively captures such instabilities:

(6) $$b_{i}^{\left(l\right)}=\frac{1}{L_{l}}\sum_{t}(d_{i}^{\left(l\right)}(t){)}^{2}.$$

This operation transforms high-frequency noise and anomalous fluctuations into stable, positive scalar features, thereby enhancing the discriminative power of the features.

#### 2.2.3. Multi-Scale Feature Concatenation and Channel Attention

Under the configuration of last-moment aggregation for approximation components and Energy Pooling for detail components, the scalar features from each decomposition level for node i are concatenated as follows:

(7) $$z_{i}=[a_{i}^{\left(1\right)},b_{i}^{\left(1\right)},\ldots ,a_{i}^{\left(L_{\max}\right)},b_{i}^{\left(L_{\max}\right)}{]}^{⊤}\in R^{H}.$$

The features of all nodes are subsequently stacked to form the feature matrix $Z\in R^{N\times H}$. When channel attention is enabled and H>1, a global attention re-weighting mechanism is applied across the scale/channel dimensions:

(8) $$w_{h}=\frac{exp(\frac{1}{N}\sum_{i=1}^{N}|Z_{i,h}|)}{\sum_{h^{\prime }=1}^{H}exp(\frac{1}{N}\sum_{i=1}^{N}|Z_{i,h^{\prime }}|)},h=1,\ldots ,H,$$

(9) $${\overset{\sim }{Z}}_{i,h}=w_{h}\cdot Z_{i,h},$$

where $w\in R^{1\times H}$ denotes the channel attention weights. This operation assigns higher weights to scales with larger average magnitudes across all nodes, thereby suppressing redundant scales and emphasizing time scales that contribute more stable and significant information.

#### 2.2.4. Integration with the Variational Graph Autoencoder

The extracted temporal feature matrix Z is subsequently utilized as the input feature matrix X for the **VGAE**. The encoder leverages **Graph Attention Layers** to fuse temporal features with the graph topology A, mapping nodes into a latent space $Z_{\mathrm{latent}}$:

(10) $$\mu ,log\sigma^{2}={Encoder}_{GAT}\left(Z,A\right),\;Z_{latent}∼N\left(\mu ,diag\left(\sigma^{2}\right)\right).$$

The training objective function L jointly constrains the structural reconstruction error, the feature reconstruction error, and the Kullback–Leibler (KL) divergence of the latent space:

(11) $$L=\lambda_{b}\cdot BCE\left(\hat{A},A\right)+\lambda_{a}\cdot MSE\left(\hat{X},X\right)-\lambda_{KL}\cdot KL\left(q∥p\right).$$

Through this **end-to-end joint training** strategy, the adaptive wavelet module effectively filters noise and extracts robust temporal patterns. This mechanism enables the graph model to prioritize nodes exhibiting significant temporal anomalous energy during structural learning, thereby enhancing the overall anomaly detection performance.

### 2.3. Spatio-Temporal Token Statistics Self-Attention

**Figure 3:**
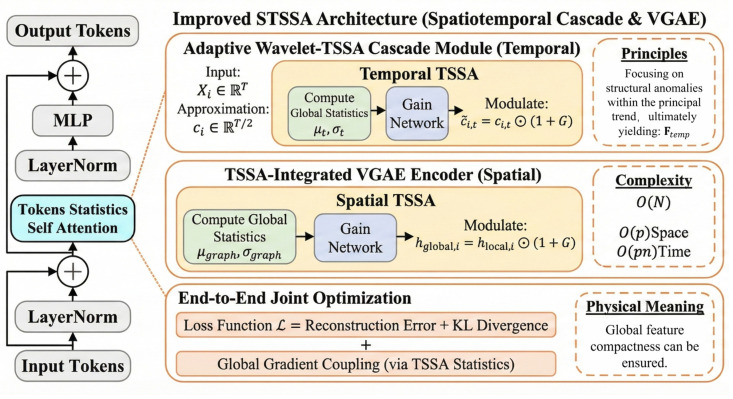
Token Statistical Self-Attention Mechanism [37].

Spatio-temporal graph data are inherently characterized by **non-stationary temporal dynamics** and **complex spatial topological dependencies**. Conventional self-attention mechanisms not only suffer from a computational bottleneck of $O(N^{2})$ but also struggle to effectively isolate multi-scale anomaly patterns in the presence of noise interference. To address these challenges, this study proposes a **cascaded spatio-temporal processing architecture** (Figure 3):

- In the temporal dimension: The architecture integrates the Adaptive Wavelet Transform with TSSA, realizing a two-stage modeling paradigm of “frequency-domain denoising followed by statistical focusing”.
- In the spatial dimension: A VGAE enhanced by TSSA is constructed, facilitating the **complementary modeling** of local topology and global statistics.

Grounded in the theory of **Variational Rate Reduction** ($MCR^{2}$), this approach reformulates the attention mechanism as an **incremental optimization process** regarding global statistics. Consequently, it explicitly enhances the **discriminative capability** of the feature space against anomaly patterns while achieving **linear complexity**.

#### 2.3.1. Variational Rate Reduction and Diagonal Approximation

The core philosophy of this study is derived from the principle of $MCR^{2}$. In the context of anomaly detection, the objective is to learn a latent representation Z such that the feature distribution of normal samples becomes maximally compact (thereby minimizing the intra-class coding rate $R_{c}$), while the overall distribution encompassing anomalies retains maximum information entropy (thereby maximizing the total coding rate R). The theoretical objective function is formalized as

(12) $$\begin{array}{c} \underset{Z}{max}\Delta R(Z,\Pi )=R(Z)-R_{c}(Z,\Pi ) \end{array}.$$

To efficiently achieve this objective within high-dimensional spatio-temporal data, we adopt a **Diagonal Covariance Approximation** strategy. This approach discards explicit subspace projection, opting instead to utilize the **first moment** (mean μ) and the **second moment** (standard deviation σ) of the features to approximate the manifold distribution. Furthermore, a non-linear gain network is employed to implicitly learn the correlations among features. The **Statistical Gating Operator** is defined as follows:

(13) $$Y=\mathrm{TSSA}(H)=H⊙\left(1+\sigma \left(\mathrm{MLP}([H\|\mu \|\sigma ])\right)\right),$$

where ‖ denotes the feature concatenation operation and σ(⋅) represents the Sigmoid activation function. The MLP is composed of linear layers and the GELU activation function.

The **physical interpretation** of this operator lies in its ability to directly contrast the local token feature H and the global statistical distribution [μ,σ]. For normal samples that **conform to** global statistical regularities, the network outputs a stable gain; conversely, for anomalous samples that **deviate from** the distribution, the network generates significant suppression or enhancement signals.

#### 2.3.2. Temporal Denoising and Statistical Focusing Strategy

The primary challenges in time-series anomaly detection lie in signal **non-stationarity** and **noise interference**. Directly applying statistical attention mechanisms to raw sequences may lead to the model **overfitting high-frequency noise**. To address this, we propose the **Adaptive Wavelet–TSSA Cascade module**, adopting a **“denoise-first, focus-later”** strategy.

Given the raw time series $x_{i}\in R^{T}$ for node i, we employ an Adaptive Wavelet Transform based on the **Lifting Scheme**. By utilizing learnable predictors and updaters to dynamically adjust the wavelet bases, the input is decomposed into **approximation components** (Approximation, $c_{i}$) and **detail components** (Detail, $d_{i}$):

(14) $$\left(c_{i},d_{i}\right)=\mathrm{AdpWavelet}\left(x_{i};\theta_{wave}\right).$$

Here, the approximation component $c_{i}\in R^{T/2}$ filters out high-frequency noise while retaining the **dominant patterns** that reflect temporal evolution trends, thereby providing a **pristine signal basis** for subsequent statistical modeling.

Subsequently, the temporal TSSA mechanism is applied to the approximation component c. The model first projects c into a high-dimensional space and then calculates the global statistics $\mu_{t}$ and $\sigma_{t}$ for each time step along the node dimension. A gain network is then employed to assess whether the trend of each node at the current moment deviates from the **collective evolutionary patterns**:

(15) $${\overset{\sim }{c}}_{i,t}=c_{i,t}⊙\left(1+G\left(c_{i,t},\mu_{t},\sigma_{t}\right)\right).$$

This design enables the model to disregard random fluctuations (filtered by the wavelet transform) and concentrate on **structural anomalies** within the major trends (captured by TSSA). Finally, robust node temporal features $F_{temp}$ are obtained through temporal aggregation.

#### 2.3.3. VGAE Integrated with TSSA

In the spatial dimension, the **VGAE** is adopted as the backbone generative framework. Conventional VGAEs rely on Graph Convolutional Networks (GCNs) for feature aggregation. While effective at utilizing local topological structures, they often lack awareness of the **global graph distribution**, making it challenging to identify nodes that are **“structurally normal but attributively anomalous.”**

To address this limitation, we embed the spatial TSSA module into the encoder hierarchy of the VGAE, constructing a **GCN-TSSA Hybrid Encoder**. Its processing workflow follows the trajectory of **“Local Aggregation, Global Enhancement, Variational Mapping”**:

(16) $$H_{\mathrm{local}}={\mathrm{GCN}}_{\mathrm{base}}(F_{\mathrm{temp}},A)\overset{\mathrm{TSSA}}{\to }H_{\mathrm{global}}\overset{{\mathrm{GCN}}_{\mu ,\sigma }}{\to }(\mu_{z},log\sigma_{z}).$$

Specifically, the first GCN layer utilizes the adjacency matrix A to aggregate local neighborhood information, generating $H_{local}$, which encapsulates local topological features. Subsequently, the spatial TSSA module computes the statistics $\mu_{graph}$ and $\sigma_{graph}$ for all nodes in the graph:

(17) $$\mu_{\mathrm{graph}}=\frac{1}{N}\sum_{i=1}^{N}h_{i},\sigma_{\mathrm{graph}}=\sqrt{\frac{1}{N}\sum_{i=1}^{N}(h_{i}-\mu_{\mathrm{graph}}{)}^{2}+\epsilon }.$$

This step enables the features of each node to be modulated by the global distribution, ensuring that anomalous nodes (where $h_{i}$ deviates significantly from $\mu_{\mathrm{graph}}$) receive a high gain. **Global statistical modulation** is then applied to $H_{local}$:

(18) $$h_{global,i}=h_{local,i}⊙\left(1+G(h_{local,i},\mu_{graph},\sigma_{graph})\right).$$

The resulting TSSA-enhanced feature matrix $H_{global}$ encompasses not only local neighborhood information but also **relative positional information** within the global graph distribution. Finally, parallel GCN layers map these features to the distribution parameters of the latent space. This architecture achieves an **organic integration** of “local topological aggregation” and “**global statistical rectification**,” significantly enhancing the discriminative power of the latent representation Z against various spatial anomalies.

#### 2.3.4. End-to-End Joint Optimization and Gradient Coupling

To achieve **synergistic gains** between feature extraction and anomaly detection tasks, the spatio-temporal TSSA module is embedded into the front-end of the VGAE, establishing an **end-to-end differentiable joint training framework**. The total loss function L is composed of reconstruction errors and a Kullback–Leibler (KL) divergence regularization term:

(19) $$\underset{\Theta }{min}L={\left\|A-A\right\|}_{F}^{2}+{\left\|X-X\right\|}_{F}^{2}+\beta \cdot D_{KL}\left(q\left(Z|X,A\right)\|p\left(Z\right)\right).$$

It is particularly noteworthy that the integration of TSSA creates a unique **Global Gradient Coupling** effect during backpropagation. In contrast to conventional networks where gradients backpropagate solely along local paths, the update of any arbitrary node feature $h_{i}$ in TSSA is constrained by global statistics:

(20) $$\frac{\partial L}{\partial h_{i}}=\underset{\mathrm{Local}\;\mathrm{Gradient}}{\underbrace{\frac{\partial L}{\partial {\tilde{h}}_{i}}}}+\underset{\mathrm{Global}\;\mathrm{Distribution}\;\mathrm{Constraiant}}{\underbrace{\sum_{j\neq i}\;\frac{\partial L}{\partial {\tilde{h}}_{j}}\cdot \left(\frac{\partial {\tilde{h}}_{j}}{\partial \mu }\frac{\partial \mu }{\partial h_{i}}+\frac{\partial {\tilde{h}}_{j}}{\partial \sigma }\frac{\partial \sigma }{\partial h_{i}}\right)}},$$

This global gradient coupling effect **compels** the model to consider the impact of updating individual node parameters on the **compactness of the global feature distribution**. Simultaneously, error gradients can backpropagate to the wavelet transform layers, driving the wavelet bases to **adaptively adjust their frequency domain responses** to retain the temporal frequency bands most beneficial for anomaly detection. This **deep synergistic mechanism** circumvents the information loss associated with multi-stage processing, realizing an **optimal solution** from raw signal processing to anomaly discrimination.

### 2.4. Anomaly Detection and Evaluation

In the testing phase, the **reconstruction error** is utilized as the anomaly scoring metric for fault detection. Specifically, for each sample window in the test set, the model computes the reconstruction output $\hat{X}$ and the reconstructed adjacency matrix $\hat{A}$. These variables are then synthesized to calculate a combined reconstruction error, serving as the anomaly score. Samples with reconstruction errors exceeding a predefined threshold are classified as anomalous, while those falling below the threshold are deemed normal.

This strategy is **predicated on the hypothesis** that, since the model is trained exclusively on normal samples, it will fail to accurately reconstruct anomalous samples, thereby yielding reconstruction errors that are significantly higher than those of normal instances. This study adopts a **composite loss function** as the anomaly scoring indicator, which consists of three components:

(21) $$L=\lambda_{b}\cdot BCE\left(\hat{A},A\right)+\lambda_{a}\cdot MSE\left(\hat{X},X\right)-\lambda_{KL}\cdot KL\left(q|p\right),$$

where $\hat{A}$ and $\hat{X}$ denote the reconstructed adjacency matrix and feature matrix, respectively.

To **circumvent the subjectivity and limitations** inherent in fixed threshold selection, this paper introduces an **adaptive threshold determination method** based on **Probability Density Estimation (PDE)**. Specifically, **KDE** is employed to model the distribution of reconstruction errors (or latent space features) derived from normal samples during the training phase. On this basis, the **decision boundary** is established according to a preset **Confidence Level**: samples located in low-density regions are identified as anomalies, whereas others are classified as normal.

Finally, to comprehensively quantify the discriminative performance of the model in anomaly detection tasks, multi-dimensional metrics including the **FAR**, the **FDR**, and the **CMR** are employed for evaluation.

## 3. Results

To systematically assess the performance of the proposed **ST-VGSAE** model in anomaly detection tasks involving graph-structured data characterized by temporal dynamics, extensive experiments were conducted utilizing the **TE** benchmark.

As a widely recognized industrial process simulation, the TEP dataset encompasses multi-dimensional sensor readings and exhibits complex temporal variations. It effectively simulates realistic industrial production environments, thereby serving as an ideal **testbed** for validating the efficacy of anomaly detection methodologies.

### 3.1. Evaluation Metrics

1. False Alarm Rate (FAR): The FAR, also known as the False Positive Rate (FPR), measures the proportion of normal samples that are erroneously classified as anomalies. It is calculated as follows:
  (22) $$\mathrm{FAR}=\frac{\mathrm{FP}}{\mathrm{FP}+\mathrm{TN}},$$
  where FP (False Positives) denotes the number of normal samples incorrectly identified as anomalies and TN (True Negatives) represents the number of normal samples correctly identified as normal.

2. Fault Detection Rate (FDR): The FDR evaluates the model’s capability to successfully detect genuine anomalous samples. Its calculation is defined as
  (23) $$FDR=\frac{TP}{TP+FN}$$
  where TP (True Positives) indicates the number of actual anomalous samples correctly identified as anomalies and FN (False Negatives) refers to the number of missed anomalous samples.

3. Correct Match Rate (CMR): To comprehensively balance the trade-off between the False Alarm Rate and the Fault Detection Rate and to evaluate the overall classification performance, the CMR is introduced. This metric is essentially equivalent to Balanced Accuracy and is calculated as
  (24) $$\mathrm{CMR}=\frac{\;(1\;-\;\mathrm{FAR})+\mathrm{FDR}}{2}$$
  Here, 1 − FAR represents Specificity (i.e., the Accuracy in identifying normal samples), while FDR represents the Accuracy in identifying anomalous samples. A higher CMR value indicates superior comprehensive performance of the model across both normal and anomalous categories.

### 3.2. Comparative Experiments

#### 3.2.1. Model Configurations

**xLSTM (Extended Long Short-Term Memory)** [38]: This model enhances the traditional recurrent neural network architecture to better capture long-range temporal dependencies in time-series data. While xLSTM improves the memory capacity for sequential information, it primarily focuses on temporal dynamics and lacks an explicit mechanism to model the spatial topological structure among multivariate sensors. Consequently, it exhibits limited robustness against high-frequency fluctuations in complex coupled systems.

**iTransformer** [39]: This architecture inverts the standard Transformer structure by embedding the entire time series of each variate as a token to learn multivariate correlations via self-attention. Although iTransformer excels in capturing global dependencies and interactions, its attention mechanism tends to be over-sensitive to local noise without robust regularization constraints. This limitation often leads to a higher False Alarm Rate (FAR) when processing industrial data with significant background noise.

**ST-VGSAE (Proposed Model)**: By integrating Spatio-Temporal Graph Attention mechanisms with Variational Graph Autoencoders, this model explicitly captures both the complex spatial correlations among sensors and multi-scale temporal dependencies. Unlike the baseline models, it utilizes variational inference to effectively suppress noise and establish a high discriminative margin, achieving a robust balance between detection sensitivity and signal stability.

#### 3.2.2. Experimental Results

**Figure 4:**
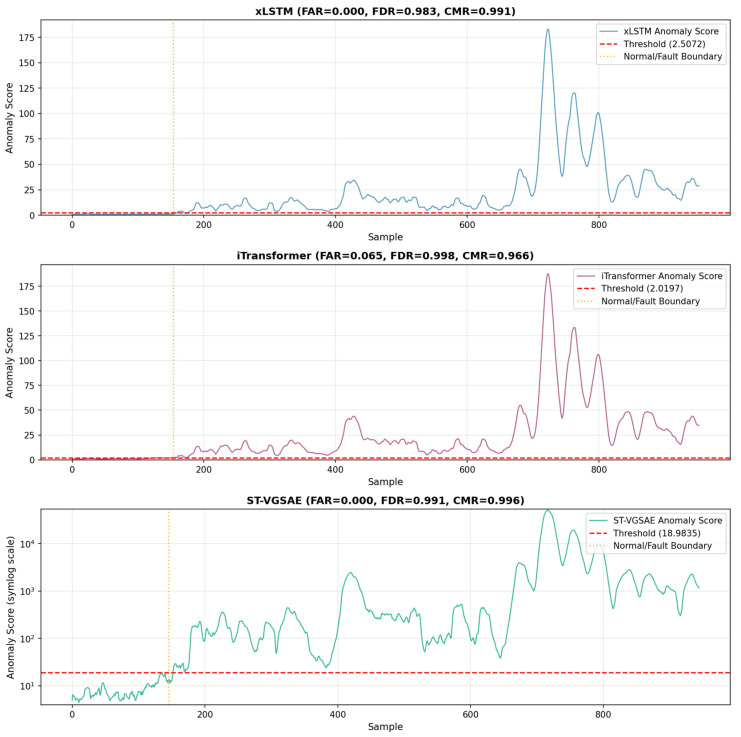
Comparative experimental results of different models.

Based on the visualization curves and quantitative metrics (FAR, FDR, and CMR), the **ST-VGSAE** model demonstrates significant advantages in fault detection accuracy, robustness, and discriminative capability (Figure 4).

1. Superior Noise Immunity and Signal Stability
  The ST-VGSAE model exhibits exceptional robustness against noise. Under normal operating conditions (samples 0–160), its anomaly score curve remains highly smooth and stable within a low-magnitude range ($10^{1}-10^{2}$), avoiding the severe sawtooth fluctuations observed in baseline models (xLSTM and iTransformer).
  Visual results indicate that xLSTM and iTransformer display obvious stochastic fluctuations in the normal region, with spurious peaks (approx. 40) appearing near sample 400. In contrast, ST-VGSAE consistently maintains a smooth, low-value state. This demonstrates that the joint constraints of the Spatio-Temporal Graph Attention Mechanism and the Variational Graph Autoencoder effectively suppress high-frequency random disturbances in industrial environments, ensuring zero false alarms (FAR = 0.000) caused by noise fluctuations.
2. Discriminative Margin with a Orders-of-Magnitude Difference
  Unlike deep temporal models that distinguish anomalies via weak distinctions on a linear scale, ST-VGSAE establishes a discriminative margin of orders of magnitude between normal and fault samples.
  Visualization on a symmetric logarithmic (symlog) scale shows that the model’s fault score peaks reach the $10^{3}$ to $10^{4}$ magnitude (max peak $\approx 5\times 10^{4}$), while the threshold is only 18.98, forming a safety boundary exceeding three orders of magnitude. Conversely, xLSTM (threshold: 2.51 and peak: 180) and iTransformer (threshold: 2.02 and peak: 190) offer a discriminative margin of only about 70 times. This suggests that the global coupling effects introduced by the ST-VGSAE significantly enhance the discriminative power of the feature space by strongly amplifying anomaly signals that deviate from the normal distribution through multi-scale feature fusion.
3. Rapid and Precise Fault Response
  The model demonstrates extremely high sensitivity and response speed to sudden faults. At the instant the fault is introduced (sample 160), the anomaly score of the ST-VGSAE exhibits a near-vertical step-like rise (jumping from $10^{1}$ directly to $10^{2}\text{–}10^{3}$), immediately breaching the control limit without detection delay or ambiguous transition zones.
  In comparison, although xLSTM and iTransformer detect the fault, their response curves show a gradual climbing process during the initial fault phase (samples 160–200), and fluctuations in the normal region obscure the fault boundary. This confirms that the deep feature extraction capabilities of the ST-VGSAE’s spatio-temporal attention mechanism and graph encoder ensure immediate capture and effective transmission of abrupt signals, achieving zero-latency fault localization.

The comparative experiments provide compelling evidence that xLSTM, constrained by its temporal memory mechanism, struggles to model complex coupled signals, resulting in significant volatility under normal conditions, and that iTransformer, while sensitive to faults, lacks effective noise suppression mechanisms, leading to a high False Alarm Rate. However, the ST-VGSAE guarantees robustness via graph-based spatial modeling and variational inference, as well as high discriminative power via spatio-temporal attention, achieving comprehensive superiority over existing mainstream methods across all key metrics.

### 3.3. Ablation Study

#### 3.3.1. Overall Performance

To investigate the contribution of individual modules to the overall system performance, we conducted ablation experiments. It is important to note that, to ensure a robust evaluation, the results presented in Table 1 represent the **average metrics calculated across all TE fault types**.

The Full Model achieved the optimal comprehensive performance, attaining a CMR of 0.9840, while simultaneously maintaining an exceptionally low False Alarm Rate (FAR: 0.0032) and a high Fault Detection Rate (FDR: 0.9711). These results strongly validate the efficacy of the proposed joint design paradigm—characterized by “temporal denoising and global statistical focusing”—in effectively handling the non-stationarity and complex topology inherent in industrial data.

#### 3.3.2. Component-Wise Ablation Analysis

1. Spatio-temporal Token Statistical Self-Attention
  The exclusion of the TSSA module resulted in a noticeable deterioration in model performance, with the CMR dropping to 0.9796 and the FDR decreasing to 0.9626. Without this module, the spatial architecture degenerates into a conventional VGAE that focuses solely on local neighborhoods. Consequently, it loses the capability to leverage the global graph distribution to modulate local features, leading to a decline in detection sensitivity towards subtle or globally distributed fault modes.
2. Adaptive Lifting Wavelet Module
  When the adaptive wavelet module is removed, the model’s False Alarm Rate (FAR) increases to 0.0054 (approximately 1.7 times that of the Full Model), and the overall CMR declines to 0.9781. In the absence of effective high-frequency noise management provided by wavelet decomposition, the model struggles to filter out normal transient fluctuations. This noise interference not only elevates the False Alarm Rate but also obscures genuine anomalous features, resulting in a lower FDR (0.9617) compared to the Full Model. This emphasizes the absolute necessity of the “denoise-first” strategy in complex temporal dynamics.

## 4. Discussion

To address the challenges posed by non-stationarity and complex topological dependencies in the monitoring of large-scale CPPS, this paper proposes an unsupervised anomaly detection framework named ST-VGSAE. First, by incorporating a learnable Adaptive Lifting Wavelet Module, this study establishes a “denoise-first, focus-later” temporal processing strategy. Experimental results demonstrate that this module dynamically adjusts operators to aggregate approximation components and pool detail components, thereby effectively filtering high-frequency noise and significantly reducing the False Alarm Rate (FAR).

Simultaneously, the proposed TSSA mechanism circumvents the computational bottlenecks of conventional attention methods. Through the global gradient coupling effect, it utilizes global statistical distributions to constrain local updates, achieving linear computational complexity while establishing a discrimination margin with an orders-of-magnitude difference.

Comprehensive validation on the TE benchmark dataset indicates that the ST-VGSAE model achieves an optimal balance between detection sensitivity and signal stability, maintaining an exceptionally low FAR while ensuring a high detection rate. Notably, its overall performance significantly outperforms state-of-the-art deep temporal baselines such as xLSTM and iTransformer. This study successfully synergizes signal processing, deep graph learning, and statistical attention theory, providing a robust, interpretable, and efficient solution for the intelligent monitoring of complex industrial processes.

## Figures and Tables

**Table 1:** Ablation study results.

Model	CMR	FAR	FDR
Only [Core Component]	0.9544	0.0063	0.9544
w/o AdpWaveletBlock	0.9781	0.0054	0.9617
w/o TSSA	0.9796	0.0032	0.9626
Full Model	0.9840	0.0032	0.9711

## Data Availability

The original contributions presented in this study are included in the article. Further inquiries can be directed to the corresponding author.
